# Brain size affects female but not male survival under predation threat

**DOI:** 10.1111/ele.12441

**Published:** 2015-05-10

**Authors:** Alexander Kotrschal, Séverine D Buechel, Sarah M Zala, Alberto Corral-Lopez, Dustin J Penn, Niclas Kolm, Gabriele Sorci

**Affiliations:** 1 Department of Zoology/Ethology, Stockholm UniversitySvante Arrhenius väg 18B. SE-10691, Stockholm, Sweden; 2 Department of Integrative Biology and Evolution, Konrad Lorenz Institute of Ethology, University of Veterinary MedicineVienna, Savoyenstraße 1a, 1160-Vienna, Austria

**Keywords:** Artificial selection, brain size, *Crenicichla*, guppy, pike cichlid, *Poecilia reticulata*, predation, semi natural, survival

## Abstract

There is remarkable diversity in brain size among vertebrates, but surprisingly little is known about how ecological species interactions impact the evolution of brain size. Using guppies, artificially selected for large and small brains, we determined how brain size affects survival under predation threat in a naturalistic environment. We cohoused mixed groups of small- and large-brained individuals in six semi-natural streams with their natural predator, the pike cichlid, and monitored survival in weekly censuses over 5 months. We found that large-brained females had 13.5% higher survival compared to small-brained females, whereas the brain size had no discernible effect on male survival. We suggest that large-brained females have a cognitive advantage that allows them to better evade predation, whereas large-brained males are more colourful, which may counteract any potential benefits of brain size. Our study provides the first experimental evidence that trophic interactions can affect the evolution of brain size.

## Introduction

Brain size variation is ubiquitous in the animal kingdom (Striedter 2005) and it is often suggested that ecologically adaptive variation in brain size is maintained by selective trade-offs. For example, larger brains enhance cognitive ability, whereas increased brain size also imposes large energetic demands that can override the cognitive benefits or even favour smaller brains (Aiello & Wheeler 1995; Kotrschal *et al*. 2013a). Several comparative studies have shown that brain size and behaviours indicative of cognitive ability are positively associated (Tebbich & Bshary 2004; Overington *et al*. 2009; Reader *et al*. 2011; MacLean *et al*. 2014). Recent experimental evidence further corroborated the link between a larger brain and improved cognitive abilities because replicated selection lines of guppies (*Poecilia reticulata*) bred for large brain size performed better in tests of cognitive ability than selection lines bred for smaller brains [Females: (Kotrschal *et al*. 2013a); Males: (Kotrschal *et al*. 2014a)]. However, brains are among the most energetically costly organs in the vertebrate body (Raichle & Gusnard 2002). The high energetic costs of brains have been shown with direct metabolic measurements (Raichle & Gusnard 2002) and there are also evolutionary trade-offs between brains and other metabolically costly tissues (Navarrete *et al*. 2011; Kotrschal *et al*. 2013a; Tsuboi *et al*. 2014). Taken together, these studies provide compelling evidence that increased brain size improves cognitive ability, but also imposes high energetic costs. For selection to favour the evolution of increased brain size, the cognitive benefits must therefore outweigh the high energetic costs (Striedter 2005). The problem is that it is unclear whether or how selection favours increased brain size. Therefore, our aim was to conduct an experiment to determine how brain size affects fitness, as part of a larger study on the evolution of brain size in guppies.

Several studies have shown that brain size is heritable [e.g. h^2^ in guppies is around 0.63 (Kotrschal *et al*. 2013a)]; however, to date, the only evidence that larger brains confer fitness benefits come exclusively from comparative studies. For example, large-brained bird species have higher survival in the wild (Sol *et al*. 2007) and they are better at colonising urban environments (Maklakov *et al*. 2011; Husby & Husby 2014) compared to small-brain species. Also, in several taxa large-brained species [mammals (Sol *et al*. 2008), birds (Sol & Lefebvre 2000), reptiles (Amiel *et al*. 2011), but not fishes (Drake 2007)] are more likely to establish viable populations after introduction events compared to small-brain species. The cognitive buffer hypothesis explains those patterns by suggesting that larger brains buffer individuals against environmental challenges by facilitating the construction of behavioural responses, which in turn increase survival (Allman *et al*. 1993; Deaner *et al*. 2003; Sol 2009). To complement studies on macroevolutionary patterns, studies on fitness within species are needed, and especially with experiments that can assess causality concerning the selective consequences due to variation in brain size. We therefore performed a test to determine how experimental changes in brain size affect survival under predation. We used guppy selection lines that had been artificially selected for either large or small relative brain size. These selection lines differ by up to 13.8% in brain size relative to body size (Kotrschal *et al*. 2014a) and, because body size does not differ between lines, they also differ in absolute brain size (Kotrschal *et al*. 2013a, 2014a). Large-brained individuals from these selection lines have been shown to outperform small-brained individuals in tests of cognitive ability (Kotrschal *et al*. 2013a,b, 2014a). Large-brained individuals of both sexes are more exploratory and show a decreased hormonal stress response (Kotrschal *et al*. 2014b). Additionally, large-brained males are more colourful than small-brained males (Kotrschal *et al*. 2015), and though colouration enhances male mating success, it also increases conspicuousness to predators (Endler 1980).

To compare the fitness consequences of selection for increased brain size, we conducted a competition study to test how large- and small-brained individuals from these selection lines differ in survival when exposed to naturalistic predation pressure. We constructed large, replicated semi-natural ‘streams’ in which we established mixed populations of marked large- and small-brained animals. In weekly censuses, we then monitored survival in the presence of a natural guppy predator, the pike cichlid (*Crenicichla alta*) until a predefined criterion of 50% survival of the populations was met. Assuming larger brains improve predator avoidance, we expected large-brained females to have higher survival under predation pressure, unless the energetic costs are over-ridden by such benefits. We had no particular prediction for males because large-brained males are more colourful, and therefore they may be more conspicuous to predators than small-brain males (Endler 1980), which may result in no advantage or a survival disadvantage.

## Methods

### Directional selection on brain mass

We examined the relationship between brain size and survival in laboratory lines of Trinidadian guppies that were artificially selected for large or small relative brain size (Kotrschal *et al*. 2013a). We used laboratory descendants of wild guppies (*P. reticulata*), whose founders (> 500 individuals) were imported in 1998 (caught in the lower regions of Quare river, Trinidad) and since then kept in large populations (> 500 individuals at any time) where they were allowed to reproduce freely. Starting in 2011, the brain size selection lines were generated using a standard bidirectional artificial selection design that consisted of two replicated treatments (three independent up-selected lines and three independent down-selected lines). Since brain size can only be quantified after dissection, we allowed pairs to breed at least two clutches before sacrificing the parents for brain quantification. We then used the offspring from parents with large or small relative brain size to breed the next generation. More specifically, to select for relative brain size, we selected on the residuals from the regression of brain size (mass) on body size (length) of both parents. We started with three times 75 pairs (75 pairs per replicate) to create the first three ‘up’ and ‘down’ selected lines (six lines in total). We summed up the male and female residuals for each pair and used offspring from the top and bottom 20% of these to form the next generation parental groups. This means we used the offspring (two males and two females) of the 15 pairs with the largest residual sums for up-selection and of the 15 pairs with the smallest residual sums for down-selection for each generation. To avoid inbreeding, full siblings were never mated. See Kotrschal *et al*. (2013a) for full details on the selection experiment. The selection lines differed in relative brain size by 9% in F2 (Kotrschal *et al*. 2013a) and in a subset of males of F3 by 13.8% (Kotrschal *et al*. 2014a), while body size did not differ between the lines. Overall, the effect of selection on brain size was not different between the sexes (Kotrschal *et al*. 2013a). Previous to the survival experiment, all fish were housed in 50-L-tanks, separated by brain size selection line, sex and replicate, containing 2 cm of gravel with a biological filter and java moss. The laboratory was maintained at 26 °C (resulting in 25 °C water temperature) with a 12 : 12 light : dark schedule. Fish were fed a diet of flake food and freshly hatched brine shrimp 6 days per week.

### The survival experiment

**Figure 1:**
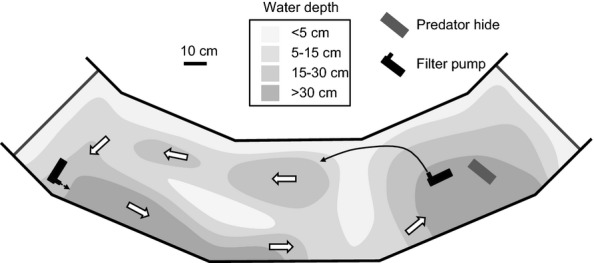
Experimental stream designed to test how brain size affects survival. Grey shadings indicate the depth profile, black arrow depicts a hose transporting water from the filter to the shallow area, white arrows indicate the direction of water flow.

The experiment was performed in a segmented glass ring tank (outer/inner diameter: 7.3/5.3 m), which we compartmentalised into six same-sized parts by inserting opaque PVC sheets, enabling us to create six replicate ‘streams’ of 3.08 m^2^ each. Our streams were based on an elegant design used by Endler (1980). Strips of filter-foam between the sides of the ring tank and the PVC sheets held the sheets in place and allowed some water flow between streams. Our aim was to recreate the natural environment of guppies in Trinidad. We, therefore filled the streams with a layer of coarse rounded (naturally) multi-coloured lime stone gravel (3–8 mm) with which we crafted areas of different depths. The created water depths ranged from 0.5 to 40 cm (gravel depth: 3–40 cm) with relatively even gradients of *c*. 30 ° between different depths. We installed two Eheim filter pumps to create filtration and water flow (2400 L × h^−1^ per pump) from the shallow to the deep area (the effective stream length from the outlet of the filter in the most shallow area to the deepest point was 4.0 m; Fig.1). The shallow areas provided refuges for the guppies in which the pike cichlid could not hunt (Endler 1980). We also placed an additional refugium [white PVC box 40 × 30 × 20 cm, gravel on bottom] with one round 10 cm wide opening in the shallowest area. The box, in which the hose that carried water from the filter from the deepest area ended, was partly submerged and fish could enter and exit it freely. We added java moss (*Taxiphyllum sp*.) and water snails (*Planorbis sp*.) as natural destruents of organic waste. Electric heaters kept the water temperature at 25 °C. Fish were fed once daily (in the morning) by scattering a near *ad libitum* ration of flake food and freshly hatched *Artemia* over the deeper areas of the stream. The amount of food was adjusted so that most food would be depleted within 3–4 min of feeding. The water current quickly dispersed the food to all areas of the streams; the flakes slowly sank to the bottom while the *Artemia* remained in the water column. Animals could thus feed from the surface, the water column and the bottom. During the feedings, we never observed any predator activity. Lighting followed a 12 : 12 schedule with bright half-hour periods of increasing and diminishing brightness, simulating dusk and dawn. During the night, faint lights simulated moon light.

**Figure 2:**
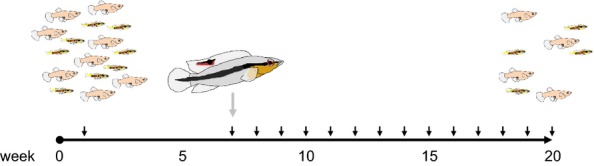
Timeline of the experimental procedure to determine the relationship between brain size and survival in guppies. The small black arrows indicate whole-population censuses, the grey arrow indicates introduction of the guppy predator, a pike cichlid. The weekly censuses stopped after a predefined 50% survival criterion was met at week 20.

At the start of the experiment (week 0), we stocked each stream with 800 fully mature, adult guppies (mean age: 220 ± 30 days, virgin and naïve to predators) balanced over sex and brain size, which had been tagged with visible implant elastomere tags 3 weeks earlier. Large-brained animals were marked with a green and red dot on the left and right body side respectively just below the dorsal fin. Small-brained animals were marked similarly but with the green dot on the right side and the red dot on the left side. The first 6 weeks following introduction, the animals were allowed to acclimatise to the novel environment. At week seven, we counted all marked fish (794 ± 2 per stream, mean 6-week survival probability per stream: 99.1%; Fig2; results section below), added one adult pike cichlid (*C. alta*, body size: 9.9–15.7 cm) per stream and stocked the deepest area of the stream with three clay pipes as shelter for the predator. *Crenicichla* are pike-like carnivores with ‘ambush and stalk’ hunting strategies, they are often sympatric to the guppy and can impose a high predation pressure (Houde 1997; Johansson *et al*. 2004). In the used setting, up to three predation events per hour per cichlid can be expected (J. Endler, personal communication). The fish were wild-caught and imported via the aquarium trade. Although the exact location of origin is unknown, and it is therefore impossible to know whether those individuals had lived with guppies in sympatry, it is safe to assume that they had foraged on small fish before (Johansson *et al*. 2004). Six weeks prior to introduction to the streams, cichlids were fed exclusively on live guppies and all individual predators consumed them readily.

We conducted weekly censuses of all marked fish until a predefined criterion was met where the last of the four subgroups (large- and small-brained males and females) reached a mean of 50% survival in all streams. This survival criterion was met at week 20 (Fig.2). At this time, fish were ca. 7 months old (mean age: 218 ± 30 days). Because one predator showed signs of stress (hiding and very little feeding) at weeks 12–15, we replaced it at week 15 with another one. The distressed predator fully recovered in its private tank. Another predator was depleting the guppy population at twice the rate than the other predators during weeks 12–15. We, therefore, food-supplemented this predator after the census at week 15 with one dead adult guppy from the pet shop every second day to keep predation rates comparable between experimental streams.

### Statistical analyses

To determine whether relative brain size influences survival under semi natural-conditions, we used two complementary approaches. First, we assessed potential differences in survival time using a proportional hazards-based mixed-effects Cox-regression model, which utilised all census data (Harrell 2001). Second, we determined the survival probability at the end of the experiment with a generalised linear mixed-effect model (GzLMM), for which we used only individual numbers at the beginning and at the end of the experiment.

#### Survival duration

As dependent variable in the Cox-regression we used individual survival (was it present/absent) at every census; as fixed factors sex (males/females), brain size selection regime (small/large) and their interaction, and as random effects we included replicate nested in stream (three replicates, two streams per replicate). We also analysed male and female survival separately, analogously to the model described above, but without sex in the model as guppies show a high degree of sexual dimorphism in colouration, size and behaviour (Houde 1997), which could potentially result in pronounced sex differences in survival. Sex-specific effects on a range of traits were also found in the brain size selection lines (Kotrschal *et al*. 2012, 2013a, 2014b). This part of the analysis was done using the ‘coxme’ package in R (Development.Core.Team, R. 2006; Therneau 2009).

#### Survival likelihood

Since most, but not all fish had survived the 7-week acclimation period before introduction of the predator (average per tank survival: 99.1%, see above and results below) and we were interested in the survival after the predator was introduced (week 7) until reaching the 50% criterion (week 20), we used a binary probit-link GzLMM to analyse survival at the end of the experiment, with the number of fish present at week 20 as dependent variable and the number of fish present at week 7 as independent variable. We used sex and brain size selection regime as fixed effects and replicate as random effect, analogously to the models described above. Similarly, we analysed survival of both sexes first in a combined model, and then in two sex-specific models. For survival in the first 6 weeks predator-free acclimation period, we used an analogous general linear mixed model (GLMM) with the number of not re-found fish as dependent variable. These analyses were done in SPSS 22.0, SPSS Inc., Chicago.

### Ethical note

Breeding and marking of experimental fish comply with the Swedish law and were approved by the Uppsala ethics committee. Animal care procedures during the predation experiment were discussed and approved by the Veterinary University of Vienna’s institutional ethics committee in accordance with good scientific practice (GSP) guidelines and national legislation.

## Results

**Figure 3:**
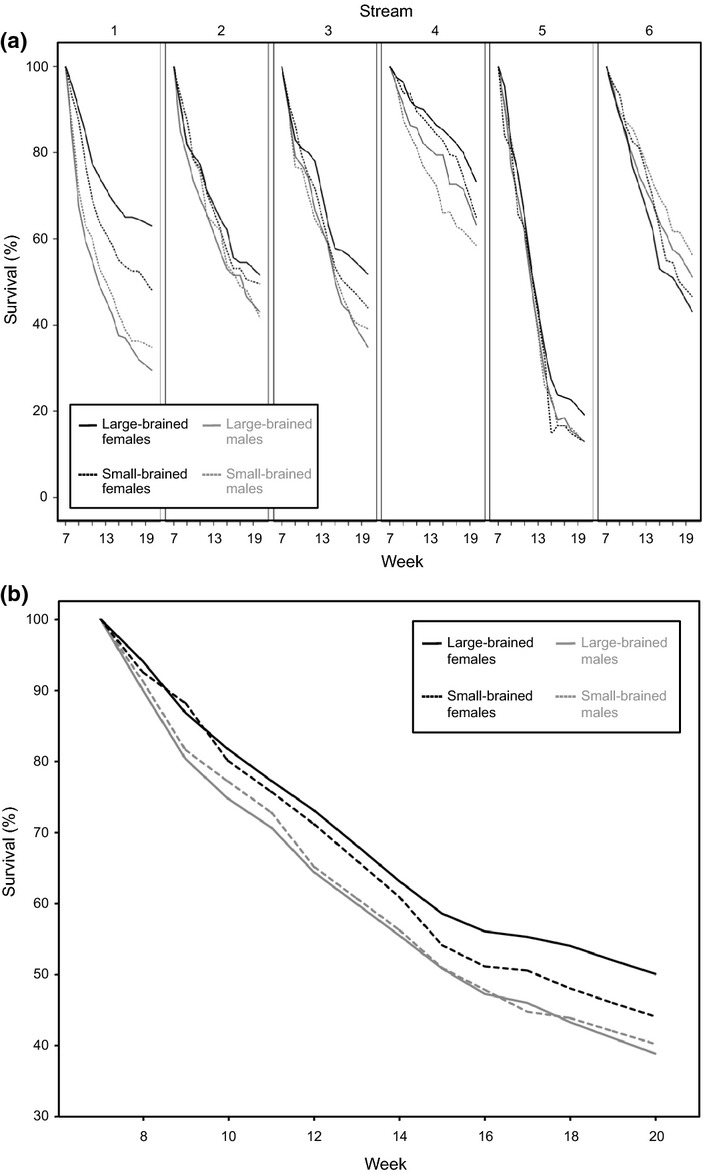
Survival curves of guppies selected for large and small relative brain size in experimental streams following introduction of a pike cichlid. (a) Shows variation in survival in the individual streams. (b) Shows the mean survival curves over all six streams. Note that in order to improve clarity, error bars are not presented. Solid lines: large-brained animals, broken lines: small-brained animals.

After the 6 weeks predator-free acclimation period, 57 individuals were not re-found and likely died of natural causes. There was no difference between individuals from large- and small-brained selection lines in the number of missing individuals, but a higher male survival. (GLMM: brain size selection regime: *F* = 0.08, *P* = 0.777; sex: *F* = 6.75, *P* < 0.019). When analysed separately, there was also no difference in initial survival between large- and small-brained females (GLMM: brain size selection regime: *F* = 0.57, *P* = 0.484). After the predators were introduced into the streams, the numbers of guppies declined steadily and the survival criterion was met for all four subgroups at week 20 (Fig.3a). Although we did not systematically observe pike cichlid hunting behaviour, our observations suggest that guppies were captured during the day by striking individuals passing by the predators’ day-roost, whereas during dusk and dawn guppies were captured by active pursuit.

### Survival duration

Overall, large-brained animals survived longer than small-brained individuals (Cox-regression: brain size selection regime: *z* = 2.12, *P *=* *0.034, sex: *z* = −1.31, *P* = 0.190, brain size selection regime × sex: *z* = −1.78, *P* = 0.074, Table1, Fig.3b). Due to our *a priory* predictions of sex-specific differences (see Methods section), we also analysed the sexes separately and found that, although large- and small-brained males did not differ in survival (Cox-regression: brain size selection regime: *z* = −0.39, *P *=* *0.700, Table1, Fig.3b), large-brained females survived on average 13.5% longer compared to small-brained females (Cox-regression: brain size selection regime: *z* = 2.19, *P* = 0.029, Table1, Fig.3b).

**Table 1:** Results of proportional hazards-based Cox-regression models investigating the influence of sex and brain size on survival duration of large- and small-brained guppies under predation pressure

	coef	Exp (coef)	SE (coef)	z	*P*
All fish					
Brain size	0.0870	1.0909	0.0410	2.12	**0.034**
Sex	−0.0533	0.9480	0.0408	−1.31	0.190
Brain size × sex	−0.1034	0.9017	0.0580	−1.78	0.074
Random effects	SD	Variance			
Stream (replicate)	0.1474	0.0217			
Stream	0.1474	0.0217			
Females					
Brain size	0.0899	1.0940	0.0410	2.19	**0.029**
Random effects	SD	Variance			
Stream (replicate)	0.0201	0.0004			
Stream	0.2256	0.0509			
Males					
Brain size	−0.0158	0.9842	0.0410	−0.39	0.700
Random effects	SD	Variance			
Stream (replicate)	0.14869	0.0221			
Stream	0.14869	0.0221			

Statistically significant results (P < 0.05) are highlighted in bold.

### Survival likelihood

The mean survival probabilities at week 20 (estimated means from a GzLMM; ± SE) were as follows: small-brained males: 40.0 ± 7.3%, large-brained males: 38.3 ± 7.2%, small-brained females: 43.6 ± 7.5%, large-brained females: 50.2 ± 7.6%. Overall, females had higher survival than males and there was a significant sex × brain size selection regime interaction (GzLMM: brain size selection regime: *F* = 2.61, *P* = 0.122; sex: *F* = 27.57, *P* < 0.001, brain size selection regime × sex: *F* = 7.82, *P* = 0.011; see the endpoints of Fig.3a and b). When analysing the sexes separately we found no difference in the numbers of large- and small-brained males that had survived until week 20 (GzLMM_males_: brain size selection regime: *F* = 0.68, *P* = 0.428). However, we found that large-brained females had on average 15.1% higher survival than small-brained females (GzLMM_females_: brain size selection regime: *F* = 10.01, *P* = 0.010).

## Discussion

Female guppies from large-brain selection lines were more likely to survive, whereas we found no survival benefits for large-brained males in our study. We suggest that enhanced predator evasion is the most likely explanation for the survival benefit for large-brained females, and below we explain why factors like ageing and some other alternatives can be ruled out. Overall, females had better survival than males, which is consistent with previous studies showing male body colouration increases their conspicuousness to predators. It is unclear why large brains did not improve male survival, and below we explain why the enhanced colouration of large-brained males likely increased their vulnerability to predation. We also discuss how our results support the hypothesis that survival under predation is an important selective force in the evolution of vertebrate brain size and the general implications of this finding.

Females from large-brain selection lines may live longer than those from short-brain lines (as a correlated trait in the selection lines), similar to mammal species with relatively larger brains typically living longer (Hofman 1993). But differences in senescence are not likely to explain our results for several reasons. First, in the few fish that died during the 6 weeks of predator-free acclimation period there was no difference between large- and small-brained females. Second, at the last census, the experimental fish were between 300 and 360 days old and guppies have a longer minimum natural life expectancy (around 400 days) in the absence of predation (Reznick 1983). Third, 95% of the parents of the experimental fish, which we keep in our laboratory for a longevity assay, were still alive when they were 1 year old [18/360 individuals (9 pairs), with no differences between groups of different brain sizes; Binomial test: large- vs. small-brained males: *P* = 0.30, large- vs. small-brained females: *P* = 0.63]. Fourth, large-brained females may be better foragers and thus survive longer. However, our near *ad libitum* feeding renders mortality due to starvation implausible. Thus, we interpret our results to be driven by predation since it is highly unlikely that the reduced survival of large-brained females was due to differences in senescence or starvation patterns between the large- and small-brained females.

The improved survival of large- over small-brained females in our study was most likely due to the cognitive improvements in large- compared to small-brain lines (Kotrschal *et al*. 2013a) that enabled them to better avoid predation. Throughout the experiment, the pike cichlids usually sat hidden in clay pipes in the deepest part of the streams, striking at fish passing by. Guppies show predator inspection (Dugatkin & Godin 1992), which may function to obtain information about the predator’s state and to demonstrate to the predator that it has been detected (Pitcher 1992). Predator inspection seems to deter predators in some cases (Godin & Davis 1995), but also increases mortality risk in others (Endler 1980; Dugatkin 1992). A change in cognitive ability may impact this behaviour in several ways. Larger brained animals may be faster at gathering and integrating information about the predator’s state and therefore inspect for a shorter time. Such improved learning abilities are thought to be key for increased survival in response to predation (Brown & Chivers 2005). Also they may remember previous inspection events for longer and therefore inspect at lower rates. Whether differences in brain size indeed relate to differences in predator inspection behaviour will be clarified in future experiments.

Females had increased survival over males in our study, and sex differences in body size, swimming ability and colouration could explain this result. Body size and swimming ability are major determinants of survival of fish in nature, such that larger (Sogard 1997) and faster swimming (Houde 1997; Plaut 2001) individuals usually survive longer under predation. In guppies, females are both considerably larger in size (Houde 1997) and able to swim faster (Kotrschal *et al*. 2014b) than males. However, better survival of larger compared to smaller brained females cannot be attributed to either of those two factors, since there are no within-sex effects in neither body size (Kotrschal *et al*. 2013a), nor swimming speed in the brain size-selected lines. Sex differences in body colouration may have also contributed to males’ lower survival, by increasing their predation risk. Conspicuous body colouration enhances male mating success, but it also increases their risk of predation (Fischer 1930; Andersson 1994; Houde 1997; Reznick *et al*. 2004). This adaptive trade-off is particularly well-documented in guppies. In his classic paper, Endler (1980) showed that introduction of a piscivorous predator fish into naturalistic ponds with guppy populations rapidly decreased the colourfulness of the males in those ponds via colour-dependent predation over two generations. Thus, male survival declined faster than females in our study, which may have been due to their being more conspicuous to predators, as well as having smaller body size and slower swimming abilities than females.

The main problem is explaining why large-brained males in our study did not have a survival advantage over small-brained males. This result was unexpected, but a recent finding provides a potential explanation. Large-brained males in these selection lines are more colourful than the small-brained males (likely due to a genetic correlation between brain size and colouration; (Kotrschal *et al*. 2015), and therefore, their increased conspicuousness to predators may have overridden the benefits of having a larger brain. Another recent study with *Drosophila melanogaster* found evidence that sexual selection can enhance cognitive performance (Hollis & Kawecki 2014), whereas our findings suggest that enhanced colouration can override the survival benefits of improved cognition (or other benefits from large brains).

Here, we experimentally tested the survival benefit of relative brain size under as natural conditions as possible. The size and topography of the streams and the species of predator closely mimicked the natural situation, while food abundance and stocking densities may be considered slightly higher. Also, in the wild, several different species may prey on guppies (Houde 1997). Arguably, the harsher conditions in the wild may even amplify any brain size-dependent survival differences if large brains enable both better predator evasion and more efficient foraging strategies.

Predation is common in guppies and other vertebrate species [examples in (Endler 1986)], and predators may drive selection for larger brains within and between vertebrate species (Kondoh 2010). Several comparative studies have found that large-brained bird species show higher survival in the wild (Dugatkin 1992; Sol *et al*. 2007) and are better at colonising urban environments (Maklakov *et al*. 2011; Husby & Husby 2014), while large-brained mammals are more likely to establish viable populations after introduction events (Sol *et al*. 2008). Variation in predation pressure may also underlie brain size variation at the within species level because it can vary between populations of the same species. For instance, in the guppy’s natural habitat, waterfalls often create natural barriers that exclude piscivorous predatory fish from areas above the waterfalls (Seghers & Magurran 1995). This reproductive isolation has led to extensive ecologically driven differentiation in morphology, behaviour and life history between fish populations inhabiting upper and lower stream areas (Reznick & Endler 1982). In the closely related Poeciliidae species *Brachyraphis episcopi*, fish from low and high predation sites differ in learning ability (Brown & Braithwaite 2005). We thus predict that such predation differences also select for differences in brain morphology.

The fact that larger brains come at a cost likely restrains the evolution towards larger brains under predation. On the individual level, the high energetic costs of brain tissue may force its bearer to forage more, thereby exposing itself for longer to predation threat (Brown 1999). This cost may be offset by a foraging advantage since larger brains can also be associated with more efficient foraging. By applying an *ad libitum* feeding regime in our artificial streams we likely reduced the potential for energy-restricted predation pressure. On the population level, known trade-offs likely restrict brain size evolution (Kotrschal *et al*. 2013a; Tsuboi *et al*. 2014). We have previously shown that individual guppies from the large-brained selection lines produce *c*. 15% fewer offspring than individuals from the small-brained lines (Kotrschal *et al*. 2013a). Therefore, small brains and higher reproduction may be successful in low-predation environments with high abundance of food, while large brains and low reproduction may be more successful in high-predation environments with lower or patchier food resources. Variation in food abundance and species interactions (i.e. predation pressure) may thus have been important ecological factors behind the remarkable variation that exists in brain size among contemporary vertebrates.

In conclusion, our study provides experimental support for the long-standing hypothesis that natural selection favours individuals with larger brains, at least under certain conditions. We suggest that a change in brain size may impact predator evasion strategies via changes in cognitive ability. Our study identifies predation pressure as a key selective pressure in the evolution of brain size in natural populations.
